# Net protein balance correlates with expression of autophagy, mitochondrial biogenesis, and fat metabolism‐related genes in skeletal muscle from older adults

**DOI:** 10.14814/phy2.14575

**Published:** 2020-10-14

**Authors:** Hexirui Wu, Jiwoong Jang, Sami Dridi, Arny A. Ferrando, Robert R. Wolfe, Il‐Young Kim, Jamie I. Baum

**Affiliations:** ^1^ Center for Human Nutrition Department of Food Science University of Arkansas Fayetteville AR USA; ^2^ Korea Mouse Metabolic Phenotyping Center Lee Gil Ya Cancer and Diabetes Institute College of Medicine, Gachon University Incheon Republic of Korea; ^3^ Center of Excellence for Poultry Science University of Arkansas Fayetteville AR USA; ^4^ Department of Geriatrics Center for Translational Research in Aging and Longevity Donald W. Reynolds Institute on Aging University of Arkansas for Medical Sciences Little Rock AR USA; ^5^ Department of Molecular Medicine College of Medicine, Gachon University Incheon Republic of Korea

**Keywords:** autophagy, muscle, protein breakdown, protein synthesis, sarcopenia

## Abstract

The mechanisms leading to sarcopenia, the main cause for frailty in older adults, are still unclear. Autophagy and the ubiquitin‐proteasome system (UPS) may play a role in mediating muscle protein breakdown related to sarcopenia. In addition to loss of muscle mass, compromised muscle performance observed in sarcopenic patients has been linked to muscle mitochondria dysfunction. Increased fat deposition and fat cell infiltration in muscle frequently seen in skeletal muscle of older adults may play an additional role for the pathogenesis of sarcopenia. Therefore, the first objective of this study was to understand differences in expression of genes related to autophagy, UPS, mitochondrial biogenesis, and fat metabolism in skeletal muscle of older adults compared with young adults. Our second objective was to determine the correlation between whole body protein kinetics (WBPK) and gene expression with age. Real‐time quantitative PCR was used to determine the relative expression of targeted genes, and hierarchical regression analysis was used to determine if age had a moderating effect on the correlation between expression of targeted genes and WBPK. Increases in the expression of autophagy‐related genes and fat metabolism‐related genes were observed in muscle of older adults compared with young adults. In addition, age enhanced the negative correlations between mitochondrial biogenesis genes and net protein balance. These results suggest that dysregulated gene expression of mitochondrial biogenesis could play a role in muscle loss in older adults.

## Graphical abstract

The objective of this study was to understand differences in expression of genes related to autophagy, UPS, mitochondrial biogenesis, and fat metabolism in skeletal muscle of older adults compared with young adults and to determine the correlation between whole body protein kinetics (WBPK) and gene expression with age.These results suggest that dysregulated gene expression of autophagy, mitochondrial biogenesis, and fat metabolism could play a role in muscle loss in older adults.

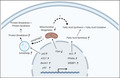

**Key Points Summary**

- Age increases expression of autophagy and fat metabolism genes in skeletal muscle.
- There was an age‐dependent negative correlation between net protein balance and mitochondrial biogenesis‐related gene expression.
- These results provide a link between changes in whole body protein kinetics (physiology) and dysregulated pathways (molecular mechanisms) in skeletal muscle of older adults.

## 1 INTRODUCTION

There are approximately 49 million adults aged 65 and older in the United States, and it is estimated that this population will grow to 98 million by 2060 (Living AfC, 2018). As we age, there is a gradual loss of skeletal muscle mass and function (Janssen, Heymsfield, & Ross, 2002), called sarcopenia (Gillespie et al., 2012; Vellas, Wayne, Romero, Baumgartner, & Garry, 1997), which may be caused by a decrease in muscle protein synthesis, an increase in muscle protein breakdown, or a combination of those responses (Baum, Kim, & Wolfe, 2016). For example, myofibrillar protein fractional synthesis rate (FSR) and sarcoplasmic protein FSR are reported to be lower in older adults compared to young adults (Cuthbertson et al., 2005). There is also a negative correlation between age and whole body amino acid kinetics (reflecting protein kinetics) such as phenylalanine and leucine flux with age, which would be consistent with accelerated protein breakdown in aging (Short, Vittone, Bigelow, Proctor, & Nair, 2004). Impairment of muscle protein synthesis has been shown to be associated with dysregulation of muscle protein synthesis‐related signaling in older adults (Guillet et al., 2004). However, no difference in muscle fractional breakdown rate has been observed between young and old adults (Fry et al., 2013). This has been further supported by additional studies demonstrating no difference in muscle protein breakdown rates between healthy young and older adults (Katsanos, Kobayashi, Sheffield‐Moore, Aarsland, & Wolfe, 2005; Volpi, Sheffield‐Moore, Rasmussen, & Wolfe, 2001). These apparently contradictory findings demonstrate the importance of gaining further understanding into the regulation of muscle protein synthesis and breakdown in older adults.

Activation of the autophagic lysosomal system is related to loss of muscle protein in catabolic states (Neel, Lin, & Pessin, 2013). However, the relation of autophagy to muscle loss with aging has not been established (White et al., 2016; Zhou et al., 2017). Autophagy is usually activated to recycle energy from intracellular damaged organelles and misfolded proteins (Levine & Klionsky, 2004). All misfolded proteins and organelles are engulfed in the autophagosome, then, fused with the lysosome. Degradation of engulfed proteins and organelles produces energy for other pathways, such as protein synthesis, to help cells overcome the crisis state (Sandri, 2010). When autophagy occurs in mitochondria, it is called mitophagy (Zhang, 2013). Increased occurrence of mitophagy indicates an increase in dysfunctional mitochondria (Kim, Rodriguez‐Enriquez, & Lemasters, 2007). It has been suggested that the accumulation of dysfunctional mitochondria in muscle leads to decreased physical performance (Drummond et al., 2014), and upregulated mitophagy decreases availability of functional mitochondria (Carter, Kim, Erlich, Zarrin‐Khat, & Hood, 2018), indicating that higher level of mitophagy or increased autophagy flux might be the potential mechanism in the development of age‐related sarcopenia in the skeletal muscle of older adults.

In addition to the possible upregulation of autophagy in sarcopenia, the ubiquitin‐proteasome pathway (UPS) may also be upregulated (Clavel et al., 2006). Two major muscle‐specific E3 ubiquitin ligases, muscle ring finger 1 (MuRF1) and muscle atrophy F‐box (MAFbx, also known as Atrogin‐1), are upregulated at mRNA level in human and rats (Clavel et al., 2006; Drummond et al., 2014) and at protein level in rats under muscle atrophy conditions (Altun et al., 2010). In addition, two genes were identified to be necessary for mediating muscle atrophy, growth arrest DNA damage‐inducible 45a (GADD45A; Baehr et al., 2016; Bullard et al., 2016) and activating transcription factor 4 (ATF4; Ebert et al., 2020; Fox et al., 2014). To date, no study compared the expression of Atrogin1, MuRF1, GADD45A, and ATF4 in skeletal muscle of older adults with those of young adults.

Another frequently reported feature of aging skeletal muscle is the imbalance between fat mass and muscle mass within skeletal muscle (Guo & Jensen, 2013). It has been previously reported that intramyocellular fat is greater in older adults when compared with their young counterparts (Hamrick, McGee‐Lawrence, & Frechette, 2016). It has been suggested that accumulated fat mass, whether infiltrated intracellularly or surrounding the muscle cells, accelerates muscle loss with age (Buch et al., 2016). Reduced fat oxidation was shown in muscle of middle‐aged adults compared with young adults (Blaak, van Baak, & Saris, 1999). It is clear that fat oxidation is reduced in skeletal muscle of older adults. However, gene expression related to fat oxidation and fat synthesis are still unknown in the skeletal muscle from older adults.

Therefore, the first objective of this study was to determine differences in gene expression related to autophagy, UPS, mitochondria, and fat metabolism in the skeletal muscle between older and young adults. Our second objective was to determine whether age has a moderating (enhancing or buffering) effect on the correlation between gene expression and whole‐body protein kinetics: protein synthesis rate (PS), protein breakdown rate (PB), and net protein balance (NB).

## 2 MATERIALS AND METHODS

### 2.1 Skeletal muscle biopsies

This study is a secondary analysis of skeletal muscle (vastus lateralis) samples from two previously published studies (Kim, Schutzler, et al., 2016; Kim et al., 2015). In total, 30 healthy adults including eleven young (18–40 years) and nineteen older adults (52–75 years) were recruited from the Little Rock area using local newspaper advertisements and flyers posted around the University of Arkansas for Medical Sciences campus and the Little Rock area. The study was conducted according to the declaration of Helsinki and a written informed consent was obtained from all participants. The study was approved by the Institutional Review Board at the University of Arkansas for Medical Sciences. Exclusion criteria is described in the original published article (Kim et al., 2015). For the current study, we analyzed the baseline muscle samples collected after an overnight fast. Characteristics of all adults are shown in Table 1.

**TABLE 1:** Participant characteristics

	Young	Old
Age	31.0 ± 5.3	65.5 ± 6.1
Female/male	7/4	9/10
Body weight, kg	82.7 ± 21.4	80.0 ± 14.8
BMI, kg/m^2^	26.0 ± 5.5	28.0 ± 3.0
LBM, kg	55.0 ± 12.3	50.0 ± 12.0*
Fat mass, kg	23.0 ± 9.3	27.8 ± 7.0*

Note

Values are expressed as mean ± *SD*.

Abbreviations: BMI, body mass index; LBM, lean body mass.

* *p* < .05.

### 2.2 Stable isotope tracer infusion and calculations of whole‐body protein kinetics

The detailed stable isotope tracer infusion protocol and calculations of protein kinetics have been described in previously published papers (Kim, Schutzler, et al., 2016; Kim et al., 2015). Briefly, after an overnight fast, two 18‐gauge catheters were placed in each lower arm, one for the stable isotope infusion and one for blood sample collection. Primed continuous infusions of L‐*ring*‐^2^H_5_‐phenylalanine (prime, 3.07 μmol/kg; rate, 5.04 μmol kg^−1^ hr^−1^) were performed. A priming dose of L‐*ring*‐^2^H_4_‐tyrosine was injected (prime, 0.44 μmol/kg) to reach isotopic equilibrium of L‐*ring*‐^2^H_4_‐tyrosine enrichment derived from L‐*ring*‐^2^H_5_‐phenylalanine infusion. The muscle biopsy used in the present study was taken 2 hr after the initiation of the tracer infusion.

Whole body PS and PB rate were calculated based on the determinations of the rate of appearance (*R*_a_) into the plasma of phenylalanine and tyrosine and the fractional *R*_a_ of endogenous tyrosine converted from phenylalanine (Kim, Suh, Lee, & Wolfe, 2016; Wolfe, 2005). Equations used for kinetic calculations were described (Kim, Suh, et al., 2016; Wolfe, 2005).

### 2.3 Quantitative real‐time PCR

RNA samples were isolated using TRIzol reagent (Invitrogen), following manufacturer instructions. Then, cDNA samples were synthesized based on the concentration of RNA samples in accordance with manufacturer instruction using Roche Lightcycler 480 system. SYBR green master mix (Quanta) was used as the reporter dye for mitochondrial biogenesis‐related genes: peroxisome proliferator‐activated nuclear receptor‐gamma (PPARγ), Peroxisome proliferator‐activated receptor gamma coactivator 1‐alpha (PGC‐1α), transcription factor A mitochondrial (Tfam), uncoupling protein 2 (UCP2), and nuclear respiratory factor 1 (NRF1); fatty acid metabolism‐related genes: carnitine palmitoyltransferase I (Cpt1b), acetyl‐CoA carboxylase (ACC), fatty acid transporter protein 1 (FATP1), fatty acid transporter 4 (FATP4), peroxisome proliferator‐activated receptor‐alpha (PPARα), sterol regulatory element binding protein 1 (SREBP1); autophagy and ubiquitin‐proteasome‐related genes: nucleoporin 62 (p62), autophagy related 3 (ATG3), autophagy related 5 (ATG5), autophagy related 7 (ATG7), microtubule‐associated proteins 1A/1B light chain 3B (LC3B), nucleoporin 53 (p53), Unc‐51 like autophagy activating kinase 1 (ULK1), Beclin1, growth arrest and DNA damage inducible alpha (GADD45A), activating transcription factor 4 (ATF4), Atrogin1, and muscle RING‐finger protein 1 (MuRF1). All primer sequences are provided in the supplementary table (Table S1). All primers were ordered from Integrated DNA Technologies. All samples and controls were analyzed in duplicate. Fold change of target genes of old group versus young group were determined using 2^−ΔΔCt^ method (Livak & Schmittgen, 2001).

### 2.4 Statistical analysis

For RT‐PCR results, multiple *t* test was used for comparing difference between young and old groups. Holm–Sidak method was used for correcting multiple comparisons (Aickin & Gensler, 1996). Statistical analyzes were performed and figures were plot in Graphpad Prism, version 6 (San Diego, CA, USA). Hierarchical linear regression analysis was performed to examine the moderating effect of age on the relations between gene expression and whole‐body protein kinetics (i.e., protein synthesis, protein breakdown, or net balance). We set BMI as a control variable in the first step (Step 1) due to its impact on protein turnover rate. Next, gene expression was entered as independent variables in the second step (Step 2). Then, we entered age as the moderator variable in the third step (Step 3). Finally, we added the two‐way interaction terms (gene × age) in the fourth step (Step 4). We confirmed that there was no multicollinearity among independent variables by examining variance inflation factors (VIF) and tolerance. Statistical analyzes were performed using IBM SPSS statistics, version 23 (Chicago, IL, USA). All significance level was set at 0.05 (*α* = 0.05). All p values that lower than 0.05 were considered significant.

## 3 RESULTS

### 3.1 Whole body protein kinetics

**FIGURE 1:**
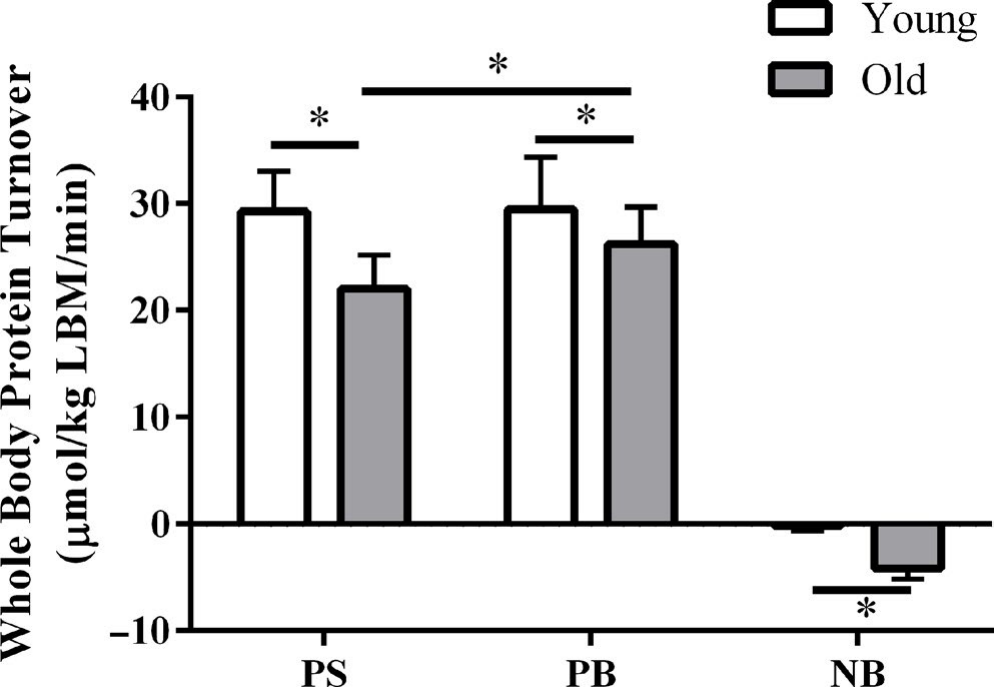
Whole body protein kinetics in skeletal muscle from young and old adults. PS, protein synthesis rate; PB, protein breakdown rate; NB, net balance; LBM, lean body mass. Values represent mean ± *SD* (young: *n* = 11, old: *n* = 19; *p < .05; Student *t* test with Holm–Sidak method)

Whole body protein kinetics (WBPK) are shown in Figure 1. Older adults showed a decrease in both PS and PB compared with young adults (*p* < .01). The NB of older adults was significantly lower than young adults (*p* < .01).

### 3.2 Autophagy gene expression, and effect of age on correlation with whole body protein kinetics

**FIGURE 2:**
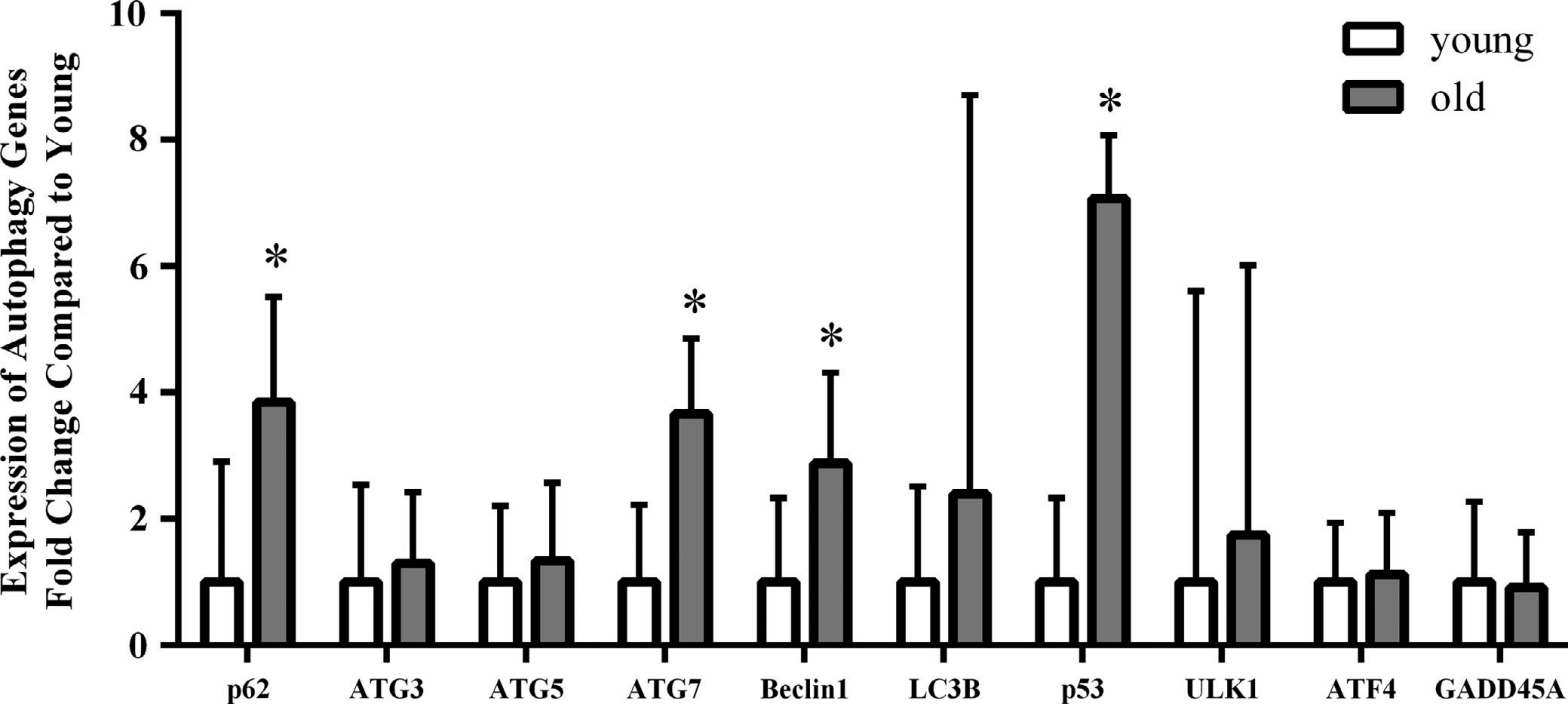
Expression of autophagy genes in skeletal muscle from young and old adults. P62, nucleoporin 62; ATG3, autophagy related 3; ATG5, autophagy related 5; ATG7, autophagy related 7; LC3B, microtubule‐associated proteins 1A/1B light chain 3B; p53, nucleoporin 53; ULK1, Unc‐51 like autophagy activating kinase 1; GADD45A, growth arrest and DNA damage inducible alpha; ATF4, activating transcription factor 4. Values represent mean ± *SD* (young: *n* = 11, old: *n* = 19; *p < .05; Student *t* test with Holm–Sidak method)

Expression of autophagy genes in older compared with young muscle is shown in Figure 2. There was an upregulation in the gene expression of p62 (*p* < .001), ATG7 (*p* < .001), Beclin1 (*p* < .01), and p53 (*p* < .001) in the older adults, compared with young adults. In the hierarchical regression analysis, we found that age has significant moderating effects on the relationship between gene expression (p53 and ULK1) and NB (Δ*R* ^2^ = 2.7% for p53 and 3.8% for ULK1, *p* < .05 for both; Table 2).

**TABLE 2:** Hierarchical regression analysis of correlation between autophagy gene expression and whole‐body protein kinetics

Autophagy	PS				PB				NB			
Variable	*B*	*SE*	*β*	Δ*R* ^2^	*B*	*SE*	*β*	Δ*R* ^2^	*B*	*SE*	*β*	Δ*R* ^2^
Step1												
BMI	0.302	0.207	0.261		0.208	0.173	0.217		0.094	0.086	0.199	
Step2				0.008				0.028				0.013
ATF4	0.114	0.232	0.094		0.175	0.191	0.175		−0.061	0.096	−0.122	
Step3				480***				0.159*				0.788***
AGE	0.203	0.037	0.718***		0.096	0.041	0.413*		0.106	0.009	0.919***	
Step4				0.006				0.010				0.000
ATF4 X AGE	0.006	0.010	0.081		0.007	0.011	0.109		−0.001	0.003	−0.023	
Step1												
BMI	0.302	0.207	0.261		0.208	0.173	0.217		0.094	0.086	0.199	.
Step2				0.020				0.042				0.005
ATG3	0.121	0.154	0.146		0.145	0.127	0.212		−0.024	0.065	−0.071	
Step 3				0.477***				0.156*				0.790***
AGE	0.202	0.037	0.715***		0.095	0.040	0.410*		0.107	0.009	0.921***	
Step4				0.016				0.040				0.008
ATG3 X AGE	0.007	0.006	0.129		0.008	0.007	0.201		−0.002	0.002	−0.089	
Step1												
BMI	0.302	0.207	0.261		0.208	0.173	0.217		0.094	0.086	0.199	.
Step2				0.003				0.012				0.006
ATG5	0.057	0.181	0.060		.0.090	0.151	0.114		−0.032	0.075	−0.82	
Step3				0.479***				0.158*				0.790***
AGE	0.202	0.038	0.717***		.0.096	0.041	0.412*		0.107	0.009	0.920***	
Step4				0.002				0.008				0.004
ATG5 X AGE	−0.003	0.008	0.050		0.005	0.009	0.093		−0.002	0.002	−0.064	
Step1												
BMI	0.291	0.213	0.25		0.202	0.178	0.210		0.089	0.089	0.187	
Step2				0.000				0.014				0.047
ATG7	0.009	0.185	0.010		0.098	0.153	0.125		−0.088	0.075	−0.228	
Step3				0.498***				0.180*				0.750***
AGE	0.208	0.038	0.739***		0.103	0.042	0.444*		0.105	0.010	0.907***	
Step4				0.020				0.026				0.000
ATG7 X AGE	0.009	0.009	0.150		0.009	0.010	0.170		0.001	0.002	0.023	
Step1												
BMI	0.287	0.210	0.255		0.186	0.0165	0.212		0.102	0.087	0.220	
Step2				0.004				0.004				0.001
Beclin1	−0.061	0.192	−0.067		−0.048	0.151	−0.067		−0.013	0.079	−0.036	
Step3				0.561***				0.243**				0.797***
AGE	0.217	0.035	0.779***		0.111	0.038	0.512**		0.106	0.009	0.929***	
Step4				0.005				0.014				0.003
Beclin1 X AGE	0.004	0.008	0.077		0.006	0.008	0.130		−0.001	0.002	−0.059	
Step1												
BMI	0.302	0.207	0.261		0.208	0.173	0.217		0.094	0.086	0.199	
Step2				0.015				0.036				0.008
GADD45A	0.129	0.194	0.123		0.169	0.160	0.194		−0.039	0.081	−0.091	
Step3				0.479***				0.158*				0.789***
AGE	0.202	0.037	0.717***		.0.096	0.040	0.412*		0.107	0.009	0.920***	
Step4				0.019				0.041				0.005
GADD45A X AGE	0.010	0.009	0.141		.0.012	0.010	0.205		−0.002	0.002	−0.068	
Step1												
BMI	0.307	0.212	0.264		0.203	0.176	0.212		0.104	0.086	0.223	
Step2				0.006				0.002				0.009
ULK1	0.021	0.051	0.074		0.010	0.043	0.045		0.010	0.021	0.093	
Step3				0.479***				0.168*				0.780***
AGE	0.208	0.037	0.732***		0.101	0.043	0.434*		0.106	0.010	0.934***	
Step4				0.007				0.040				0.038*
ULK1 X AGE	−0.001	0.002	−0.090		−0.003	0.002	−0.210		0.001	0.000	0.204*	
Step1												
BMI	0.302	0.207	0.261		0.208	0.173	0.217		0.094	0.086	0.199	
Step2				0.006				0.012				0.001
LC3B	0.069	0.165	0.082		0.083	0.137	0.119		−0.014	0.069	−0.039	
Step3				0.485***				0.162*				0.787***
AGE	0.204	0.037	0.721***		0.097	0.041	0.418*		0.106	0.010	0.919***	
Step4				0.007				0.017				0.004
LC3B X AGE	0.004	0.007	0.088		0.006	0.008	0.139		−0.001	0.002	−0.065	
Step1												
BMI	0.302	0.207	0.261		0.208	0.173	0.217		0.094	0.086	0.199	
Step2				0.003				0.015				0.011
P62	0.041	0.129	0.061		0.071	0.107	0.128		−0.030	0.054	−0.108	
Step3				0.489***				0.167*				0.779***
AGE	0.205	0.037	0.726***		0.099	0.041	0.424*		0.106	0.010	0.916***	
Step4				0.022				0.045				0.005
P62 X AGE	0.006	0.005	0.151		0.007	0.006	0.217		−0.001	0.001	−0.068	
Step1												
BMI	0.302	0.207	0.261		.0.208	0.173	0.217		0.094	0.086	0.199	
Step2				0.019				0.003				0.054
P53	−0.086	0.113	−0.139		−0.027	0.095	−0.052		−0.060	0.046	−0.234	
Step3				0.462***				0.157*				0.741***
AGE	0.201	0.038	0.714***		0.097	0.042	0.416*		0.105	0.009	0.904***	
Step4				0.002				0.001				0.027*
P53 X AGE	0.002	0.006	0.049		−0.001	0.007	−0.035		**0.003**	0.001	0.191*	

Note

Step 1 = control variable; Step 2 = independent variable; Step 3 = moderator variable; Step 4 = interaction terms; *B* = unstandardized regression coefficient; *SE* = standard errors for unstandardized regression coefficient; *β* = standardized regression coefficient; Δ*R* ^2^ = changes in *R* ^2^; PS = protein synthesis rate; PB = protein breakdown rate; NB = protein net balance; BMI = body mass index; ATF4 = activating transcription factor 4; ATG3 = autophagy related 3; ATG5 = autophagy related 5; ATG7 = autophagy related 7; Beclin1 = mammalian ortholog of yeast Atg6; GADD45A = growth arrest and DNA damage inducible alpha; ULK1 = unc‐51 like autophagy activating kinase 1; LC3B = microtubule associated protein 1 light chain 3 beta; p62 = ubiquitin‐binding protein p62; p53 = tumor protein p53.

* *p* < .05;

** *p* < .01;

*** *p* < .001.

### 3.3 Ubiquitin‐proteasome system gene expression, and effect of age on correlation with whole body protein kinetics

**FIGURE 3:**
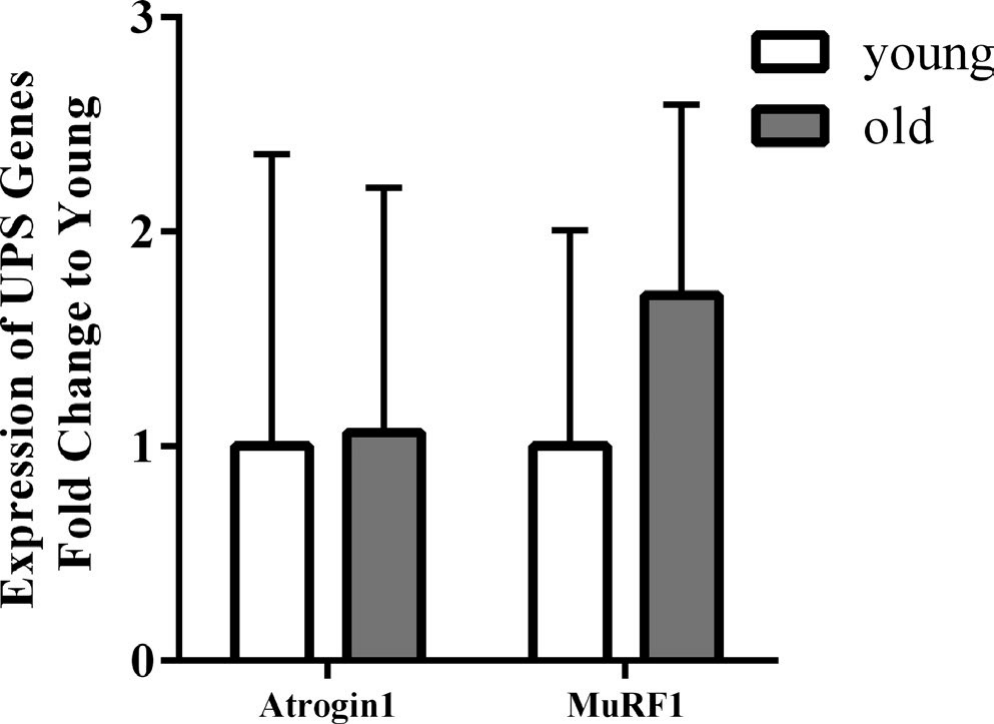
Expression of UPS genes in skeletal muscle from young and old adults. Atrogin1, muscle atrophy F‐box; MuRF1, muscle RING‐finger protein 1. Values represent mean ± *SD* (young: *n* = 11, old: *n* = 19; Student *t* test with Holm–Sidak method)

Expression of ubiquitin‐proteasome system (UPS) genes in older compared with young muscle is shown in Figure 3. MuRF1 and Atrogin‐1 showed no significant difference between older and young adults. In the hierarchical analysis, we found no moderating effects of age on the relationship between any of UPS gene expression and dependent variables (Table 3).

**TABLE 3:** Hierarchical regression analysis of correlation between UPS gene expression and whole‐body protein kinetics

UPS	PS				PB				NB			
Variable	*B*	*SE*	*β*	Δ*R* ^2^	*B*	*SE*	*β*	Δ*R* ^2^	*B*	*SE*	*β*	Δ*R* ^2^
Step1												
BMI	0.302	0.207	0.261		0.208	0.173	0.217		0.094	0.086	0.199	
Step2				0.010				0.001				0.096
Atrogin‐1	−0.090	0.165	−0.099		0.026	0.138	0.034		−0.115	0.065	−0.311	
Step3				0.471***				0.167*				0.726***
AGE	0.203	0.038	0.718***		0.099	0.042	0.427*		0.103	0.009	0.891***	
Step4				0.016				0.020				0.001
Atrogin‐1 X AGE	−0.007	0.007	0.129		0.007	0.008	0.144		0.001	0.002	0.023	
Step1												
BMI	0.291	0.213	0.250		0.202	0.178	0.210		0.089	0.089	0.187	
Step2				0.001				0.008				0.067
MuRF1	−0.037	0.221	−0.032		0.089	0.184	0.093		−0.126	0.089	−0.267	
Step3				0.488***				0.175*				0.739***
AGE	0.206	0.039	0.731***		0.102	0.042	0.438*		0.104	0.009	0.900***	
Step4				0.007				0.009				0.000
MuRF1 X AGE	0.007	0.011	0.089		0.006	0.012	0.099		0.001	0.003	0.018	

Note

Step 1 = control variable; Step 2 = independent variable; Step 3 = moderator variable; Step 4 = interaction terms; *B* = unstandardized regression coefficient; *SE* = standard errors for unstandardized regression coefficient; *β* = standardized regression coefficient; Δ*R* ^2^ = changes in *R* ^2^; PS = protein synthesis rate; PB = protein breakdown rate; NB = protein net balance; BMI = body mass index; atrogin‐1 = muscle atrophy F‐box protein; MuRF1 = muscle ring‐finger protein‐1.

* *p* < .05;

*** *p* < .001.

### 3.4 Differential mitochondrial gene expression, and effect of age on correlation with whole body protein kinetics

**FIGURE 4:**
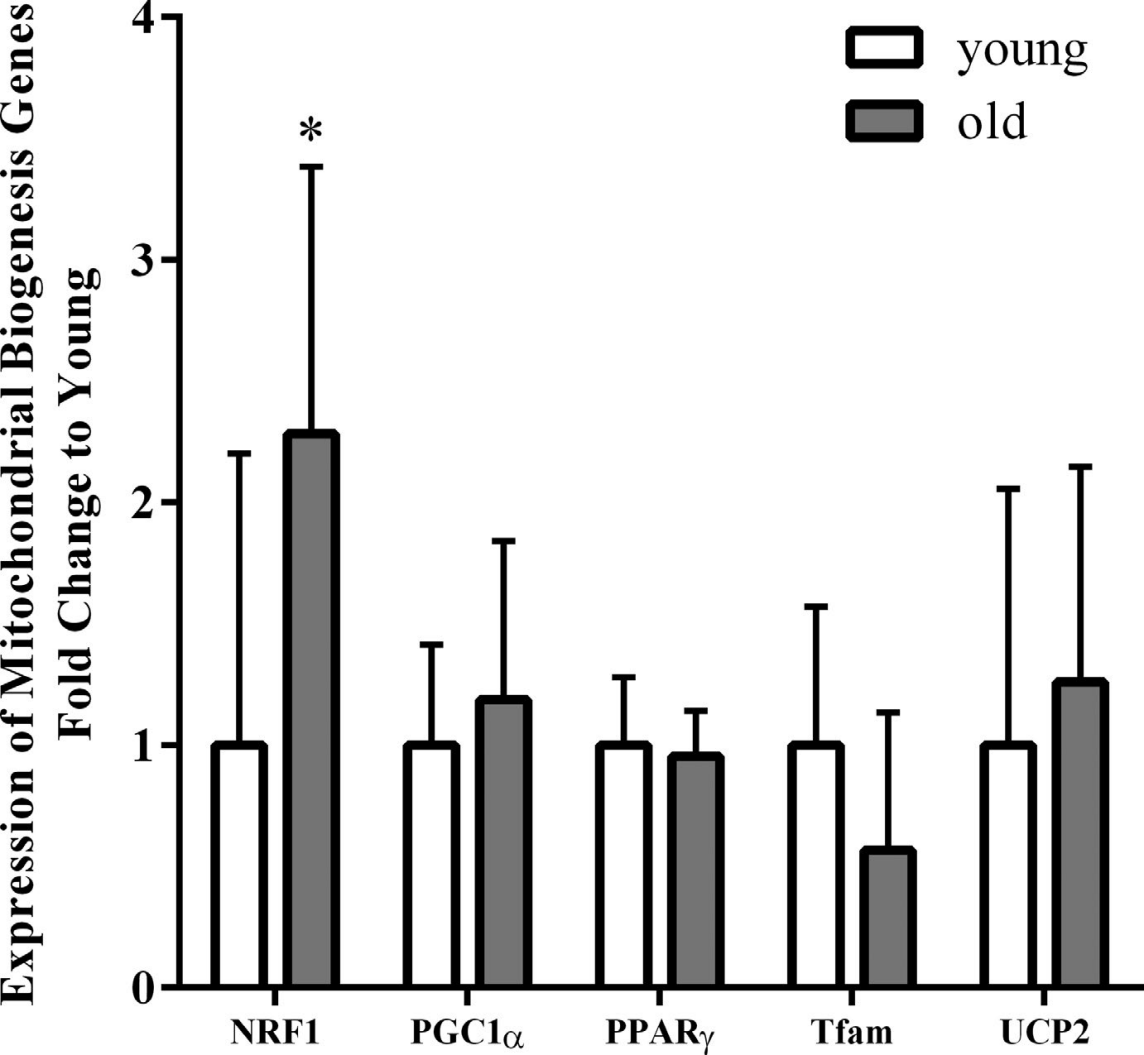
Expression of mitochondrial biogenesis genes in skeletal muscle from young and old adults. PPARγ, peroxisome proliferator‐activated nuclear receptor‐gamma; PGC‐1α, peroxisome proliferator‐activated receptor gamma coactivator 1‐alpha; Tfam, transcription factor A mitochondrial; UCP2, uncoupling protein 2; NRF1, nuclear respiratory factor 1. Values represent mean ± *SD* (young: *n* = 11, old: *n* = 19; *p < .05; Student *t* test with Holm–Sidak method)

Expression of mitochondrial genes in older versus young muscle is shown in Figure 4. NRF1 was higher in the older adults (*p* < .01) compared with young adults. In the hierarchical analysis, we found that age has a significant moderating effect on the relationship between NRF1 gene expression and NB (Δ*R* ^2^ = 6.1%, *p* < .01; Table 4).

**TABLE 4:** Hierarchical regression analysis of correlation between mitochondrial biogenesis gene expression and whole‐body protein kinetics

Mitochondrial biogenesis	PS				PB				NB			
Variable	B	SE	β	ΔR^2^	B	SE	β	ΔR^2^	B	SE	β	ΔR^2^
Step1												
BMI	0.302	0.207	0.261		0.208	0.173	0.217		0.094	0.086	0.199	
Step2				0.293**				0.133*				0.345***
NRF1	0.352	0.098	0.543**		0.195	0.092	0.366*		0.156	0.040	0.589***	
Step3				0.203**				0.046				0.444***
AGE	0.172	0.048	0.609**		0.068	0.053	0.291		0.104	0.012	0.900***	
Step4				0.027				0.006				0.061**
NRF1 X AGE	−0.023	0.017	−0.445		−0.009	0.020	−0.209		−0.014	0.004	−0.671**	
Step1												
BMI	0.302	0.207	0.261		0.208	0.173	0.217		0.094	0.086	0.199	
Step2				0.011				0.042				0.026
PGC1α	0.155	0.275	0.103		0.256	0.225	0.205		−0.100	0.113	−0.162	
Step3				0.485***				0.164*				0.781***
AGE	0.204	0.037	0.721***		0.098	0.040	0.419*		0.106	0.009	0.915***	
Step4				0.006				0.007				0.000
PGC1α X AGE	−0.011	0.018	−0.089		−0.010	0.020	−0.100		−0.011	0.004	−0.016	
Step1												
BMI	0.302	0.207	0.261		0.208	0.173	0.217		0.094	0.086	0.199	
Step2				0.002				0.012				0.015
PPARγ	0.026	0.118	0.041		0.058	0.098	0.113		−0.032	0.049	−0.126	
Step3				0.491***				0.170*				0.775***
AGE	0.206	0.037	0.729***		0.100	0.041	0.429*		0.106	0.101	0.916***	
Step4				0.016				0.035				0.005
PPARγ X AGE	0.005	0.005	0.127		0.006	0.005	0.190		−0.001	0.001	−0.072	
Step1												
BMI	0.302	0.207	0.261		0.208	0.173	0.217		0.094	0.086	0.199	
Step2				0.092				0.109				0.006
Tfam	0.465	0.2066	0.303		0.417	0.220	0.330		.0.048	0.0116	0.076	
Step3				0.415***				0.114*				0.795***
AGE	0.193	0.038	0.682***		0.083	0.041	0.357*		0.109	0.009	0.944***	
Step4				0.017				0.010				0.015
Tfam X AGE	−0.020	0.019	−0.143		−0.012	0.021	−0.108		−0.007	0.005	−0.133	
Step1												
BMI	0.302	0.207	0.261		0.208	0.173	0.217		0.094	0.086	0.199	
Step2				0.024				0.028				0.002
UCP2	0.114	0.132	0.160		0.101	0.110	0.172		0.013	0.055	0.043	
Step3				0.473***				0.154*				0.786***
AGE	0.201	0.037	0.712***		0.095	0.041	0.407*		0.106	0.010	0.919***	
Step4				0.009				0.007				0.005
UCP2 X AGE	−0.006	0.008	−0.133		−0.004	0.009	−0.114		−0.002	0.002	−0.095	

Note

Step 1 = control variable; Step 2 = independent variable; Step 3 = moderator variable; Step 4 = interaction terms; *B* = unstandardized regression coefficient; *SE* = standard errors for unstandardized regression coefficient; *β* = standardized regression coefficient; Δ*R* ^2^ = changes in *R* ^2^; PS = protein synthesis rate; PB = protein breakdown rate; NB = protein net balance; BMI = body mass index; NRF1 = nuclear respiratory factor 1; PGC1α = peroxisome proliferator‐activated receptor gamma coactivator 1‐alpha; PPARγ = peroxisome proliferator‐activated receptor gamma. Tfam = mitochondrial transcription factor A; UCP2 = uncoupling protein 2.

* *p* < .05;

** *p* < .01;

*** *p* < .001.

### 3.5 Fatty acid metabolism gene expression in young and older adults, and effect of age on correlation with whole body protein kinetics

**FIGURE 5:**
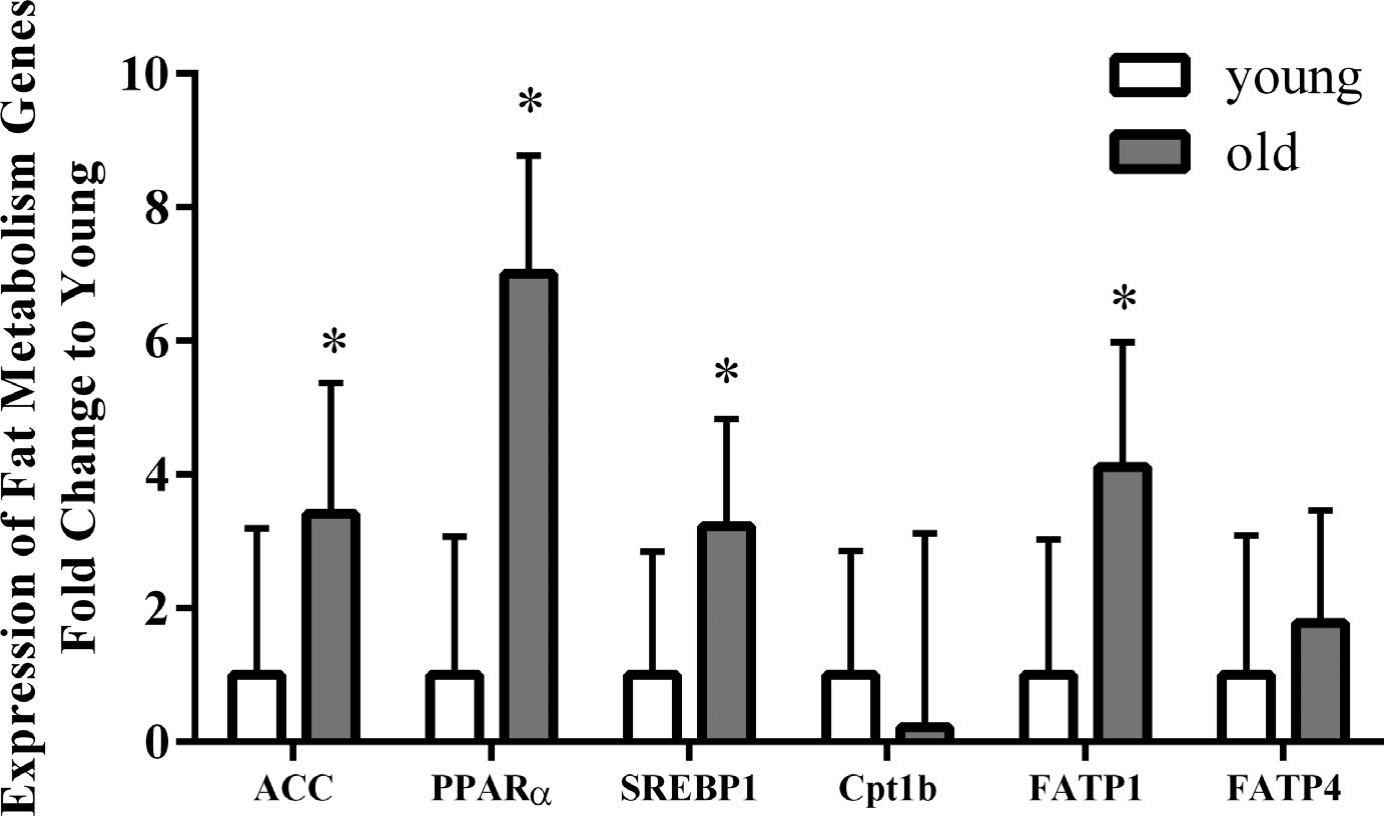
Expression of fat metabolism genes in skeletal muscle from young and old adults. Cpt1b, carnitine palmitoyltransferase I; ACC, acetyl‐CoA carboxylase; FATP1, fatty acid transporter 1; FATP4, fatty acid transporter 4; PPARα, peroxisome proliferator‐activated nuclear receptor‐alpha; SREBP1, sterol regulatory element binding transcription factor 1. Values represent mean ± *SD* (young: *n* = 11, old: *n* = 19; *p < .05; Student *t* test with Holm–Sidak method)

Expression of fatty acid metabolism genes in old versus young muscle is shown in Figure 5. FATP1, an insulin‐sensitive fatty acid transporter, was upregulated in the older adults compared with young adults (*p* < .001). The expression of the adipogenesis related gene, PPARα (*p* < .001), was significantly higher in the older adults compared with young adults. The fatty acid biogenesis‐related genes, SREBP1 (*p* < .01) and ACC (*p* < .01), were upregulated in the older adults. In the hierarchical analysis, we found that age has a significant moderating effect on the relationship between Cpt1 gene expression and PS (Δ*R* ^2^ = 6.3%, *p* < .05) or PB (Δ*R* ^2^ = 13.3%, *p* < .05; Table 5).

**TABLE 5:** Hierarchical regression analysis of correlation between fat metabolism gene expression and whole‐body protein kinetics

Fat metabolism	PS				PB				NB			
Variable	*B*	*SE*	*β*	Δ*R* ^2^	*B*	*SE*	*β*	Δ*R* ^2^	*B*	*SE*	*β*	Δ*R* ^2^
Step1												
BMI	0.302	0.207	0.261		0.208	0.173	0.217		0.094	0.086	0.199	
Step2				0.024				0.043				0.002
Cpt1	‐−0.072	0.084	−0.159		−0.080	0.070	−0.215		0.008	0.036	0.045	
Step3				0.475***				0.155*				0.790***
AGE	0.202	0.037	0.714***		0.095	0.040	0.408*		0.107	0.009	0.921***	
Step				0.063*				0.133*				0.015
Cpt1 X AGE	−0.015	0.007	−0.375*		−0.018	0.008	−0.544*		0.003	0.002	0.181	
Step1												
BMI	0.302	0.207	0.261		0.208	0.173	0.217		0.094	0.086	0.199	
Step2				0.009				0.017				0.001
FATP1	0.061	0.120	0.097		.0.071	0.100	0.136		−0.010	0.050	−0.037	
Step3				0.484***				0.162*				0.787***
AGE	0.203	0.037	0.720***		0.097	0.041	0.417*		0.106	0.010	0.919***	
Step4				0.014				0.029				0.003
FATP1 X AGE	0.005	0.005	0.119		0.006	0.006	0.172		−0.001	0.001	−0.056	
Step1												
BMI	0.302	0.207	0.261		0.208	0.173	0.217		0.094	0.086	0.199	
Step2				0.012				0.024				0.002
FATP4	0.078	0.128	0.115		.0.090	0.106	0.160		−0.012	0.054	−0.043	
Step3				0.482***				0.161*				0.787***
AGE	0.203	0.037	0.719***		.0.097	0.041	0.415*		0.106	0.010	0.919***	
Step				0.016				0.032				0.003
FATP4 X AGE	0.006	0.006	0.130		0.007	0.006	0.184		−0.001	0.001	−0.053	
Step1												
BMI	0.215	0.271	0.175		0.163	0.230	0.157		0.052	0.100	0.116	
Step2				0.056				0.005				0.239*
PPARα	−0.232	0.214	−0.257		−0.057	0.186	−0.075		−0.174	0.071	−0.528	
Step3				0.471***				0.227*				0.603***
AGE	0.223	0.051	0.776***		0.131	0.056	0.539*		0.092	0.011	0.879***	
Step4				0.047				0.099				0.017
PPARα X AGE	−0.021	0.014	−0.317		−0.025	0.016	−0.458		0.005	0.003	0.192	
Step1												
BMI	0.302	0.207	0.261		0.208	0.173	0.217		0.094	0.086	0.199	
Step2				0.006				0.017				0.007
SREBP1	0.055	0.133	0.078		.0.079	0.110	0.137		−0.024	0.055	−0.084	
Step3				0.488***				0.166*				0.782***
AGE	0.205	0.037	0.724***		0.098	0.041	0.422*		0.106	0.010	0.917***	
Step4				0.018				0.041				0.006
SREBP1 X AGE	0.006	0.006	0.138		0.007	0.006	0.206		−0.001	0.001	−0.078	
Step1												
BMI	0.302	0.207	0.207		0.208	0.173	0.217		0.094	0.086	0.199	
Step2				0.020				0.038				0.002
ACC	0.086	0.110	0.301		0.097	0.091	0.202		−0.012	0.046	−0.049	
Step3				0.486***				0.163*				0.786***
AGE	0.204	0.037	0.722***		0.098	0.040	0.419*		0.106	0.010	0.918***	
Step4				0.009				0.019				0.002
ACC X AGE	0.004	0.005	0.098		0.004	0.005	0.140		−0.001	0.001	−0.044	

Note

Step 1 = control variable; Step 2 = independent variable; Step 3 = moderator variable; Step 4 = interaction terms; *B* = unstandardized regression coefficient; *SE* = standard errors for unstandardized regression coefficient; *β* = standardized regression coefficient; Δ*R* ^2^ = changes in *R* ^2^; PS = protein synthesis rate; PB = protein breakdown rate; NB = protein net balance; BMI = body mass index; Cpt1 = carnitine palmitoyltransferase 1; FATP1: fatty acid transport protein 1; FATP4 = fatty acid transport protein 4; PPARα = peroxisome proliferator‐activated receptor alpha; SREBP1 = sterol regulatory element‐binding transcription factor 1; ACC = acetyl‐CoA carboxylase.

* *p* < .05;

*** *p* < .001.

## 4 DISCUSSION

**FIGURE 6:**
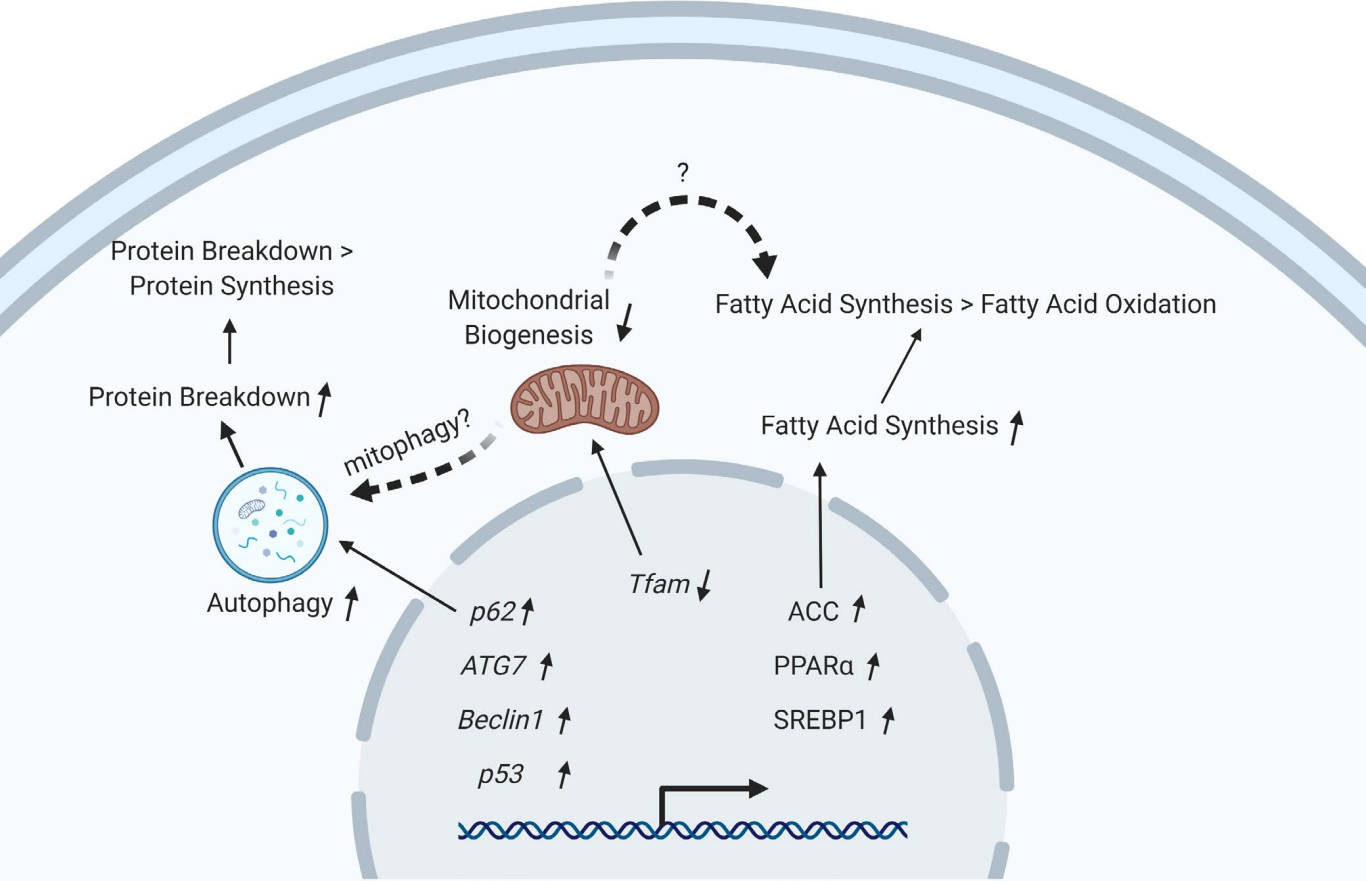
Schematic diagram showing dysregulated pathways in the skeletal muscle from older adults. Upregulated expression of p62, ATG7, Beclin1, and p53 may contribute to increased autophagy to further increase protein breakdown and mitophagy, which may be why a decrease in net protein balance was observed in the skeletal muscle of older adults. Decreased number of mitochondria may be caused by increased mitophagy in older adults

To our knowledge, this is the first study to demonstrate that the expression of genes involved in the regulation of autophagy, UPS, mitochondrial biogenesis, and fat metabolism are differentially regulated in the skeletal muscle of young versus older adults. In addition, this is the first study, to our knowledge, to examine the correlation between age, WBPK, and expression of genes related to protein synthesis, protein breakdown, and energy metabolism (Figure 6).

In the present study, markers of autophagy such as p62, ATG7, Beclin1, and p53, were upregulated in the skeletal muscle of older adults compared with young adults. This is supported by studies in skeletal muscle taken from older adults showing upregulated expression of p62 (Nogalska, Terracciano, D'Agostino, King Engel, & Askanas, 2009), and aging mice had higher cytosolic expression of p62 and Beclin1 (Sakuma et al., 2016). Consistent with the mice study (Sakuma et al., 2016), we found no differences in LC3B expression in older adults compared with young adults. One group of researchers showed that overexpression of GADD45A in the tibialis anterior muscle of healthy mice developed significant muscle atrophy (Bullard et al., 2016). However, we found no difference in expression of GADD45A and ATF4 between young and older adults. This discrepancy could be explained by the difference between artificial skeletal muscle atrophy in model animal and age‐related skeletal muscle dysfunction in humans. Interpreting our data in the context of the results reported by Sakuma et al (Sakuma et al., 2016), we suggest that autophagy could be dysregulated, especially upregulation of p62 and Beclin1. Over‐expression of these genes would facilitate the formation of autophagosome in the skeletal muscle in aged individuals, which could contribute to muscle loss. At the same time, the gene expression patterns of GADD45A and ATF4 under aging scenario need more investigation. Notably, a discrepancy exists between upregulated autophagy gene expression and decreased PB in older adults, compared with young adults. There are other multiple potential reasons for decreased PB in older adults: (a) physiologically, autophagy flux is affected by formation of autophagosome, fusion of autophagosome and lysosome, and the activity of lysosomal enzymes; (b) besides autophagy and UPS contribute to protein breakdown, calpain Ca^2+^‐dependent cysteine proteases also regulate protein breakdown; (c) the PB demonstrated here is a whole body protein breakdown rate, however, the pattern of muscle protein breakdown rate could be different with whole body protein breakdown rate.

E3 ubiquitin ligase is universally accepted as the central regulator of the UPS process (Ardley & Robinson, 2005). Overexpressed E3 ubiquitin ligase results in over‐activation of UPS and unnecessary protein degradation (Milan et al., 2015). Atrogin‐1 (Gomes, Lecker, Jagoe, Navon, & Goldberg, 2001) and MuRF1 (Bodine et al., 2001) are two well‐established skeletal muscle specific E3 ubiquitin ligases, which have been shown to be highly upregulated in mice with muscle atrophy (Tando et al., 2016). In addition, Atrogin‐1 and MuRF1 expression were upregulated in sedentary men who underwent 48 hr of unloading by unilateral lower limb suspension, a procedure to induce muscle disuse‐related atrophy (Reich, Chen, Thompson, Hoffman, & Clarkson, 2010). Another in vivo model of age‐related muscle loss showed downregulated expression of Atrogin‐1 and MuRF1 in the gastrocnemius muscle (Edstrom, Altun, Hagglund, & Ulfhake, 2006). In this study, we did not find differences in expressions of either Atrogin‐1 or MuRF1 between young and older adults. However, differences in inducing skeletal muscle loss in the animal models (such as dietary restriction‐induced atrophy vs. age‐related atrophy) could explain the contradictory results in the expression patterns of Atrogin‐1 and MuRF1.

Mitochondrial dysfunction has been widely reported in the skeletal muscle of older adults. Pre‐frail older adults have lower rates of ATP production, lower abundance of mitochondrial respiratory complex I, IV, and V and lower enzymatic activity of complex I, II, and IV compared to healthy active older adults (Andreux et al., 2018). Similarly, reduced cytochrome c oxidase (COX) activity and decreased expression of muscle specific PGC1α and COX I have been observed in low‐functioning older adults (Joseph et al., 2012). In the present study, we found that expression of PGC1α and PPARγ, two important transcription factors mediating mitochondrial biogenesis, did not differ between young and older adults. NRF1 expression was higher in skeletal muscle from older adults versus younger adults. These findings are supported by Lezza et al. (2001) who found an increase in NRF1 gene expression in skeletal muscle from older versus young adults. However, this same study also found an increase in Tfam gene expression with age (Lezza et al., 2001), where our results showed no difference in Tfam gene expression between older and young adults.

Increased fat deposition in aging skeletal muscle has been frequently reported (Tardif et al., 2014). It was previously shown that ACC, the rate‐limiting enzyme for fatty acid synthesis, was upregulated in the skeletal muscle of older adults, which is in line with our findings. In addition, PPARα is well known for mediating the activation of adipocyte differentiation during adipogenesis (Goto et al., 2011). Here, we also found upregulated levels of PPARα gene expression in skeletal muscle from older adults. Therefore, we suggest that elevated fat deposition could be the results of enhanced fatty acid synthesis and adipogenesis activation caused by ACC and PPARα upregulation. FATP1 and FATP4, two skeletal muscle abundant fatty acid transport proteins, were reported to be differentially expressed in the muscle of exercised healthy young adults (Jeppesen et al., 2012). In another in vivo model of muscle hypertrophy using myostatin knockout mice, density of muscle FATP1 and FATP4 proteins were lower than wild‐type (Baati et al., 2017). These studies suggest FATP1 and FATP4 are upregulated with muscle growth. However, we found that FATP1 and FATP4 are upregulated in the skeletal muscle of older adults. But notably, despite the fact that upregulated FATP1 and FATP4 levels would suggest higher fatty acid transport capacity for oxidation, unchanged gene expression of Cpt1b, the rate‐limiting enzyme for fatty acid oxidation, between young and old adults in our study would explain the deficiency in fatty acid catabolism reported elsewhere. As a transcription factor that regulates fatty acid synthesis in lipogenic tissues, overexpression of SREBP1 was also shown to induce muscle atrophy and decreases protein synthesis rate in an in vitro model of human skeletal muscle (Dessalle et al., 2012). Consistent with this, we found upregulated SREBP1 levels in the skeletal muscle of older adults.

It was present that obese adults had significantly lower whole body and muscle protein synthesis rate versus lean counterparts (Guillet et al., 2009). However, this study did not measure whole body and muscle protein breakdown rate, in which net balance between protein synthesis and breakdown was unknown. Another study measured WBPK showed no difference in net balance between lean and obese adults (Gougeon et al., 2008). In present study, we also did not observe any difference in net balance between normal weight and overweight and obese adults. Hierarchical regression analysis revealed that autophagy, UPS, mitochondrial biogenesis, and fat metabolism‐related genes were not correlated with WBPK (PS, PB, and NB) after introducing BMI as moderator. Therefore, we concluded that these gene expressions could not be used as predictor for WBPK.

There are several limitations to this study. First, we only measured the gene expression of selected genes between two age groups, and further validation of gene expression via immunoblotting was not conducted. Any difference found on the transcription level might not be reflective of the protein level or enzymatic activity. Second, we only determined the correlations between WBPK and expression of genes related to selected pathways, and the correlations do not completely reflect the causality. In addition, we only compared the differences in WBPK between young and older adults. Previously, Volpi et al. (Volpi et al., 2001) reported that muscle protein fractional synthesis rate was not different between young and old adults. However, muscle protein breakdown rate was not measured in this study. Finally, there are additional genes which could have been measured in this study related to the proteasome pathway, such a NEDD4, TRIM32, and Fbxo30, but were not measured due to limited sample availability.

Taken together, this is the first study to examine the correlation between skeletal muscle gene expression related to protein breakdown, mitochondrial biogenesis, fat metabolism, and whole‐body protein kinetics in young versus older adults. However, further research is needed to define the mechanisms that link WBPK to skeletal muscle function in aging adults.

## CONFLICT OF INTEREST

The authors have no conflicts of interest to disclose.

## AUTHOR CONTRIBUTIONS

JIB and RRW conceptualized the idea for the project. HW and SD conducted the gene analysis. RRW, I‐YK, and AAF designed, implemented, analyzed, and reported the initial data from the human muscle biopsy studies. HW had primary responsibility for writing the first draft of the manuscript and making the figures. JJ and I‐YK conducted the correlation analysis and had primary responsibility for making the tables. All authors had input in the study interpretation and conclusions. All authors reviewed and approved the manuscript before submission.

## Supporting information



Table S1

## Data Availability

All raw data may be requested from the corresponding authors via email. Contact J.I.B. for gene analysis data and I‐Y.K. for correlation analysis data.
