# Supplementary material for: Net protein balance correlates with expression of autophagy, mitochondrial biogenesis, and fat metabolism‐related genes in skeletal muscle from older adults
Source: Physiol Rep. 2020 Oct 14;8(19):e14575. doi: 10.14814/phy2.14575 (PMC7556313; doi:10.14814/phy2.14575)
Supplement: Supplementary file 1 — Table S1 [file PHY2-8-e14575-s001.docx]

**Supplemental Table 1**

##### Sequences of primers used for real-time PCR

| Gene Name | Forward Sequence (5’ – 3’) | Reverse Sequence (5’ – 3’) |
| --- | --- | --- |
| Autophagy |  |  |
| P62 | GAGCGGCTCTGGACACCAT | GTGGGCAAAAGTGGTCACAA |
| P53 | TGCAATAGGTGTGCGTCAGAA | CCCCGGGACAAAGCAAA |
| ATF4 | CAGACGGTGAACCCAATTGG | CAACCTGGTCGGGTTTTGTT |
| GADD45A | GATGTGGCTCTGCAGATCCA | ATGTCGTTCTCGCAGCAAAA |
| Beclin1 | CAAGATCCTGGACCGTGTCA | CCTGGGCTGTGGTAAGTAATGG |
| LC3B | GGCGCTTACAGCTCAATGCT | TGCTGTGTCCGTTCACCAA |
| ULK1 | AAAGCGAATTTTGTGTGATTTCC | CCCAACAATTCCAAAGGTTTATTT |
| ATG3 | GGGCCGGCCGCTACT | CCAGTGCCTTTCCCTTCACA |
| ATG5 | AAACCCATTCCTTCCAAGCTAGT | GCCAGGGACCACAGTGAAA |
| ATG7 | AGCAGCCCACAGATGGAGTAG | ACGGTCACGGAAGCAAACA |
| UPS |  |  |
| Atrogin1 | AAGGTAGCGGGTGTGTATTATGC | TCATGGGAAAGGGTATGTGAATC |
| MuRF1 | CAACCTGTGCCGGAAGTGT | CTGGTCCAGTAGGGATTTGCA |
| Mitochondrial Biogenesis |  |  |
| PPARγ | GACCACTCCCACTCCTTTGA | GATGCAGGCTCCACTTTGAT |
| PGC1α | GGAACTGCAGGCCTAACTCC | CACTGTCCCTCAGTTCACCG |
| NRF1 | CCACAGGCAGATGAATGTCTTG | TCCTGGGAAGGAGAGGAGATG |
| Tfam | ATGCTTATAGGGCGGAGTGG | TGGTTTCCTGTGCCTATCCA |
| UCP2 | TCAGTGCTGGTGGAGTTGACA | GGGATCCTGGCTGGTACGA |
| Fat Metabolism |  |  |
| PPARα | GACCACTCCCACTCCTTTGA | GATGCAGGCTCCACTTTGAT |
| ACC | CATTAGCACAGACATACCT | CACCAATACTCACTTCACT |
| SREBP1 | GTATCAGGCAACTCACTAC | AACATCCATCACTCAACAG |
| FATP1 | TCTTCTGGTCACTACTCA | CCTCGCTCTGTAATCATAA |
| FATP4 | CCTGTTGTTCCTCTACTT | CCTGATGGTCTTGATGAA |
| Cpt1 | TGAGCGACTGGTGGGAGGAG | GAGCCAGACCTTGAAGTAGCG |
| GAPDH | ATGGGGAAGGTGAAGGTCG | GGGGTCATTGATGGCAACAATA |
